# An mHealth App (eSkinHealth) for Detecting and Managing Skin Diseases in Resource-Limited Settings: Mixed Methods Pilot Study

**DOI:** 10.2196/46295

**Published:** 2023-06-14

**Authors:** Rie R Yotsu, Diabate Almamy, Bamba Vagamon, Kazuko Ugai, Sakiko Itoh, Yao Didier Koffi, Mamadou Kaloga, Ligué Agui Sylvestre Dizoé, Kouamé Kouadio, N’guetta Aka, Luc Kowaci Gontran Yeboue, Koffi Aubin Yao, Ronald E Blanton

**Affiliations:** 1 Department of Tropical Medicine Tulane School of Public Health and Tropical Medicine New Orleans, LA United States; 2 School of Tropical Medicine and Global Health Nagasaki University Nagasaki Japan; 3 Department of Dermatology National Center for Global Health and Medicine Shinjuku Japan; 4 Department of Dermatology Université Alassane Ouattara Bouaké Côte d’Ivoire; 5 Raoul Follereau Institute Côte d’Ivoire Adzopé Côte d’Ivoire; 6 Department of Genome Informatics Graduate School of Medicine Osaka University Suita Japan; 7 Department of Home Health and Palliative Care Nursing Graduate School of Health Care Sciences Tokyo Medical and Dental University Tokyo Japan; 8 Centre Suisse de Recherches Scientifiques en Côte d’Ivoire Abidjan Côte d’Ivoire; 9 National Buruli Ulcer Control Program Ministry of Health of Côte d’Ivoire Abidjan Côte d’Ivoire; 10 National Control Program for Elimination of Leprosy Ministry of Health of Côte d'Ivoire Abidjan Côte d’Ivoire; 11 Pasteur Institute Abidjan Côte d’Ivoire; 12 Hope Commission International Abidjan Côte d’Ivoire

**Keywords:** dermatology, developing countries, digital health tool, LMICs, low- and middle-income countries, skin, teledermatology, eHealth application, skin disease, digital health intervention, health platform, system usability

## Abstract

**Background:**

In sub-Saharan Africa, the disease burden from skin diseases, including skin-related neglected tropical diseases (skin NTDs), is extremely high. These diseases often are overlooked due to limited access to health care stemming from, for example, remote geographical locations and a lack of experts. To address these gaps, we developed a mobile health app, eSkinHealth, which is a field-adapted platform to serve as a portable electronic patient chart and for teledermatology.

**Objective:**

The purpose of the study is to evaluate the usability and effectiveness of the app in rural Côte d’Ivoire for diagnosing and managing skin NTDs and other skin diseases.

**Methods:**

A 2-arm trial with local health care providers and patients with skin diseases was implemented over a 3-month period. The providers were assigned to an intervention receiving the eSkinHealth app or control with usual care. Four nurses and 8 community health care workers participated in each arm. The training was provided on the use of the app to the intervention arm only, while both arms were trained on skin diseases. For the usability study, we evaluated our approach with the System Usability Scale (SUS) and in-depth interviews. For the effectiveness study, our primary outcome was to evaluate the detection and management of 5 skin NTDs as our targeted diseases, namely, Buruli ulcer, leprosy, lymphatic filariasis, scabies, and yaws, using the eSkinHealth app. Procedures of our methods were reviewed and approved by the institutional review board of the Ministry of Health and by Tulane University.

**Results:**

The mean age of our participants (providers) was 40.5 and 42.5 years for the intervention and control arms, respectively, and all were male (n=24). The average SUS scores taken from the intervention arm at baseline, the midpoint (6 weeks), and the end of study (12 weeks) were 72.3 (SD 11.5), 72.3 (SD 12.4), and 86.3 (SD 10.8), respectively. All participants interviewed, including 4 dermatologists and program managers, were satisfied with the app. Especially community health care workers felt empowered by being equipped with the tool. A total of 79 cases of skin NTDs were reported in the intervention arm as compared to 17 cases in the control arm (*P*=.002). Besides the skin NTDs, more skin diseases and conditions were reported from the control than from the intervention arm (*P*<.001). However, 100 cases (66%) were not given any particular diagnosis in the control arm and were documented only as a “dermatosis.” In the intervention arm, 151 cases (72.9%) were diagnosed within the eSkinHealth platform, and the remaining were diagnosed on-site by dermatologists.

**Conclusions:**

The study provided evidence for the usability and effectiveness of the eSkinHealth app embedded into our surveillance approach to improve the detection and management of skin NTDs and other skin diseases in Côte d’Ivoire and, furthermore, is expected to contribute to knowledge on mobile health approaches in the control of skin diseases in resource-limited settings.

**Trial Registration:**

ClinicalTrials.gov NCT05300399; https://clinicaltrials.gov/ct2/show/NCT05300399

## Introduction

The prevalence of skin diseases is extremely high in sub-Saharan Africa [1-7]. These diseases are most often overlooked due to a lack of local specialists and a lack of experience among Western specialists looking at darker skin [8,9]. Sometimes, it is also because of geographical barriers to accessing health care or the health-seeking behavior of patients and their families that they do not usually visit health care facilities for skin lesions [8]. However, if left untreated, even some of the most common skin diseases could have severe complications (eg, scabies could lead to rheumatic fever and nephropathy, as well as often debilitating physical, social, and mental effects that may deprive one of educational and social opportunities) [10,11]. Furthermore, some diseases, including leprosy, Buruli ulcer, lymphatic filariasis, and other skin infections, lead to lifelong disabilities and deformities if not diagnosed and treated early [10,12]. These skin infections that prevail in low- and middle-income countries (LMICs) are members of the skin-related neglected tropical diseases (skin NTDs) listed by the World Health Organization (WHO) and targeted for disease control globally. Concurrently, more than 1 billion individuals worldwide are known to be affected or at risk for skin NTDs [13]. In particular, recently, integration within the skin NTDs has been promoted in order to strengthen the disease control of this set of diseases [14-16]. The health system built upon this approach is also expected to benefit not only skin NTDs but other skin diseases that are prevalent in LMICs [13,14].

Observation of the skin could be very informative. Without undergoing invasive examinations requiring special skills and equipment, many skin diseases could be diagnosed with just a sufficient patient history and examination of the skin. This is well suited to field settings in LMICs. Photos of the skin lesions could serve as an alternative to direct observation and, if of sufficiently good quality, could allow for the diagnosis to be made on-site or remotely. Telemedicine for dermatology, or teledermatology, is currently an emerging field taking advantage of this unique feature of skin diseases. A few attempts at teledermatology have been made in sub-Saharan African countries and have shown promising results [17-19]. These previous efforts have faced a number of challenges that we plan to overcome with the proposed work, which has the following features:

- A field-adapted mobile health (mHealth) app that could provide direct diagnostic and management assistance to health care workers in a remote setting: There is no novel tool to support teledermatology, especially in LMICs where internet accessibility and connection quality are challenges. It is also of note that photos alone are often not adequate to make a correct diagnosis, as outlined previously by Resneck et al [20]. Although clinical photos offer essential information, they need to be accompanied by some clinical information to make a more accurate diagnosis. Furthermore, such a tool needs to be optimized for use on the skin of people of color, given that the diagnosis of several skin conditions on skin type IV and darker remains a challenge [21,22].
- A platform for storage of longitudinal patient records for improved follow-up: We have been conducting active surveillance for skin diseases in Côte d’Ivoire [2]. After providing a diagnosis, the patients need to be followed up, especially as most skin diseases are chronic in nature; this is an ethical obligation in medicine. However, there is a lack of skills and expertise in dermatology to pursue this, which is a universal situation in most LMICs [23]. Our field is no exception, and it has been challenging to follow up with our patients without making repeated field visits, which are often a long distance from city centers [24]. In addition, this lack of capability to follow up with patients is partly due to a lack of a system to document patient records. A platform that stores serial photo documentation of the clinical course could guide health care practitioners, both on-site and remotely, to provide better care.
- A platform for the formal collection and automatic organization of clinical and image data of the skin: Currently, teledermatology is mostly done on platforms without any formal framework for the collection or organization of data [19,25]. There is a need for developing such a platform both for direct patient care and epidemiologic purposes, especially for the organization of clinical photos, which is a cumbersome task if done manually. Patient information management is another challenge in developing a successful teledermatology system, ensuring that patient privacy is fully protected. Nowadays, social networking sites such as WhatsApp and Facebook are sometimes used for teledermatology [19,25-27], but these informal platforms need to be used with care, considering patient privacy. If there is a platform that addresses these gaps, this could further support data analysis and quality control.

In summary, with targeted training, a technology-assisted decision support system, and a telemedicine network, local health care workers could be leveraged to enhance the diagnosis and management of these conditions as well as support health care managers in quality control. If an mHealth app that overcomes these current gaps and weaknesses is developed, this could serve as a breakthrough in managing skin diseases in LMICs.

This project is built upon a previous project for the development of a prototype smartphone or tablet app for skin diseases, which we named the eSkinHealth app [28]. This app is aimed at on-site and remote diagnosis, monitoring, clinical decision support, and geographic mapping of skin diseases adapted for use in LMICs and for skin type IV and darker. Through the lifecycle of this project, we aim to develop a powerful and comprehensive but easy-to-use mHealth app that could be used for the diagnosis and management of all types of skin conditions, especially focused for use in LMICs.

## Methods

### Study Design

We conducted a pilot trial with 2 arms over a 3-month follow-up in rural villages in Côte d’Ivoire, where multiple skin NTDs are coendemic. A mixed methods approach was used to evaluate the usability and effectiveness of the eSkinHealth app in the early detection and case management of skin diseases. We selected 5 skin NTDs, namely Buruli ulcer, leprosy, lymphatic filariasis, scabies, and yaw, as our primary target diseases to evaluate our approach.

### Recruitment Procedure

We selected 8 primary health centers (PHCs) in Sinfra Health District with multiple skin NTD coendemicities as our study sites. Sinfra Health District is located in a central district of the country, with a population of 283,971 in 2021. We selected the PHCs based on the number of cases of our target skin NTDs in the past 3 years, the population size of the catchment area, geographical distribution, and access. Using the information, we matched the PHCs (primarily those with similar sizes) and divided them equally into intervention and control arms. Targeted patients included individuals with skin conditions who accessed the selected PHCs for diagnosis and treatment of their conditions.

### Participant Inclusion or Exclusion Criteria

Two groups of eligible participants were defined as below.

#### Eligible Patients With Skin Conditions

Eligible patients in selected PHCs were defined as those who were clinically suspected or diagnosed with skin NTDs (Buruli ulcer, leprosy, lymphatic filariasis, scabies, and yaws) or other skin conditions. Patients were excluded if they were unable to provide consent for the study or reside outside of the target site.

#### Eligible Health Care Providers

Eligible local health care providers included nurses or community health care workers (CHWs) who were 18 years or older, working at PHCs or within the catchment area of the selected PHCs in Côte d’Ivoire, able to read and speak fluent French, willing to participate in the pilot study for the 3-month study duration and use a provided tablet with the eSkinHealth app if they were assigned to the intervention arm, and able to consent to participate in the study. Ineligible local health care providers were defined as those who were planning to leave the job within the study period and had difficulty operating mobile devices.

A total of 4 nurses and 8 CHWs were selected to participate in the study for each arm. One nurse per PHC was selected, while for CHWs, the number was chosen based on the population of the catchment area of the PHC. All participants were formally enrolled only after signing the informed consent form.

### Intervention

The eSkinHealth app has 2 main functions: to serve as a portable electronic medical record and a platform for teledermatology when users are in need of support from a remote specialist. The current prototype of the eSkinHealth app is made up of 6 primary screens: patient ID and demographics, symptom list, basic symptom information, clinical notes, photo list, and evolution list [28]. The content of these screens has been developed based on our previous field surveys [2] and the research team’s experience in managing dermatological patients. We have built-in some special features within each screen. They are as follows:

1. The “patient ID and demographics” screen allows for the entry of basic background information, including age, sex, occupation, and educational level. Automatic calculations are embedded for BMI and mid-upper arm circumference, allowing assessment of the patient’s nutritional status.
2. Patients may present with multiple skin conditions, and these could be organized and managed under the “symptom list” screen. When a diagnosis is entered, it appears on this screen.
3. In the “basic symptom information” screen, important information needed in diagnosing a skin disease is provided in a drop-down menu, so it is not missed and is easy to enter.
4. Physical examination results of a 1-day visit could be entered in the “clinical notes” screen. One of the unique functions that we included here is the itchiness and pain visual scales, whereby patients themselves could touch the screen and indicate how they are experiencing these sensations each time.
5. Clinical photos could be taken, which are automatically organized in the “photo list” screen.
6. Lastly, if patients are to return to the clinic, the evolution of their condition could be seen at a glance in the “evolution list” screen, allowing comparison with previous presentations.

This app is not a disease-specific tool and is fit for use with a wide range of skin conditions. Other special features of the app include patient information security using QR codes. Patients are issued a QR code on their first visit. When they present it to a local health care provider with a device running the app during follow-up visits, it allows access to their records, making their health records secure and portable.

**Figure 1:**
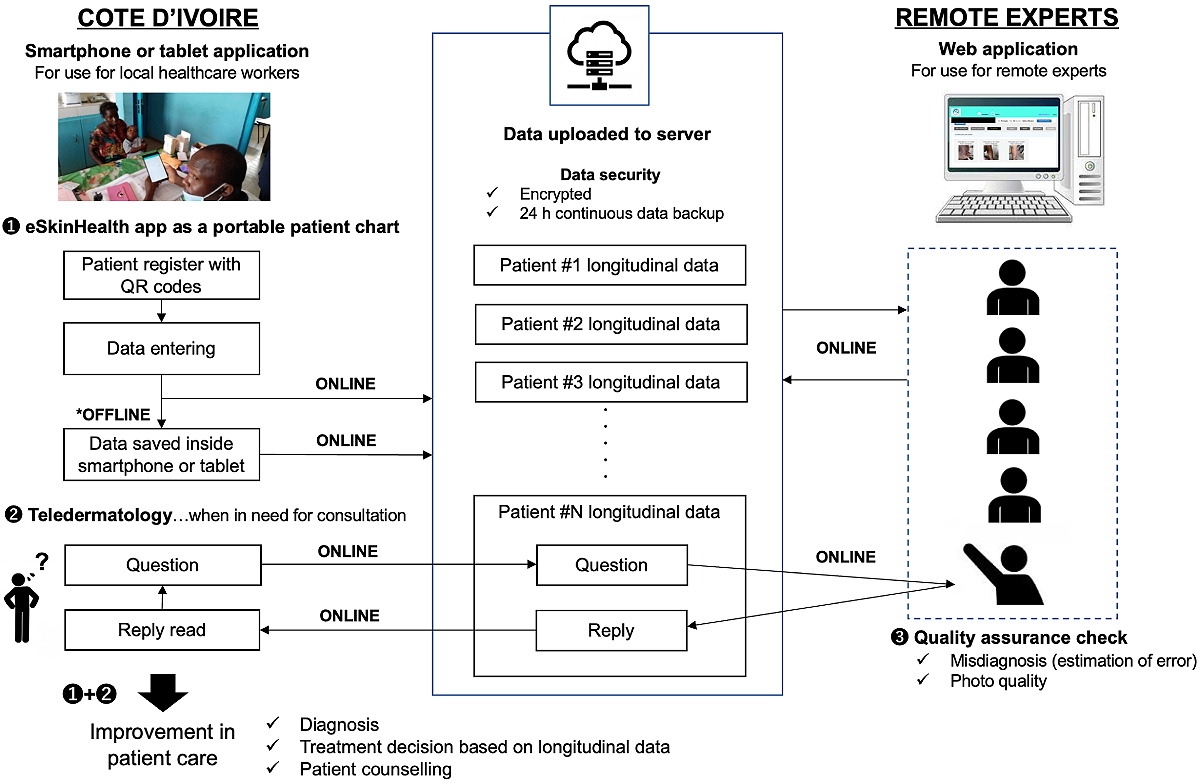
Data flow of the eSkinHealth app.

The eSkinHealth app is currently available for Android tablets with an OS version above 9.0. In the project, we used the Lenovo Tab M10 FHD Plus ZA5V0274JP. In addition, we have a web-based platform for the app to manage the case and facilitate a consultation with a remote health care provider. The app does not require a constant internet connection. Patient visits can be conducted, and all necessary information can be entered offline. When the users have access to the internet, the app synchronizes with the platform via our database server, transmitting and retrieving data (Figure 1). The platform includes the following additional functionalities: data overview, search function, and graphical display of data.

### Usability Study

We conducted a 3-month pilot trial to evaluate the usability of eSkinHealth. Local health care providers in the intervention arm were provided with a tablet with eSkinHealth installed and a Wi-Fi router and were trained on how to use the app by study staff. All local health care providers, irrespective of intervention or control arm, were provided with training on the screening and management of important skin diseases, including our target 5 skin NTDs. We then applied a questionnaire survey to investigate the usability of the eSkinHealth app [29]. As for usability, the questionnaire surveys used the System Usability Scale (SUS), developed and validated by Brooke [30,31]. We used the French-language version of SUS (Multimedia Appendix 1). Furthermore, we conducted several semistructured, in-depth interviews with:

- Users (ie, local health care providers) to gather feedback on their experience, perceived value, and willingness to use the eSkinHealth app and identify the obstacles and challenges faced in the implementation of the mobile apps; and
- Dermatologists and program managers to collect and further examine their opinions about the feasibility, advantages, and disadvantages of adopting the eSkinHealth app by local health care providers already involved in the delivery of primary health care.

Topic guidelines for these interviews are provided in Multimedia Appendix 2.

### Effectiveness Study

**Figure 2:**
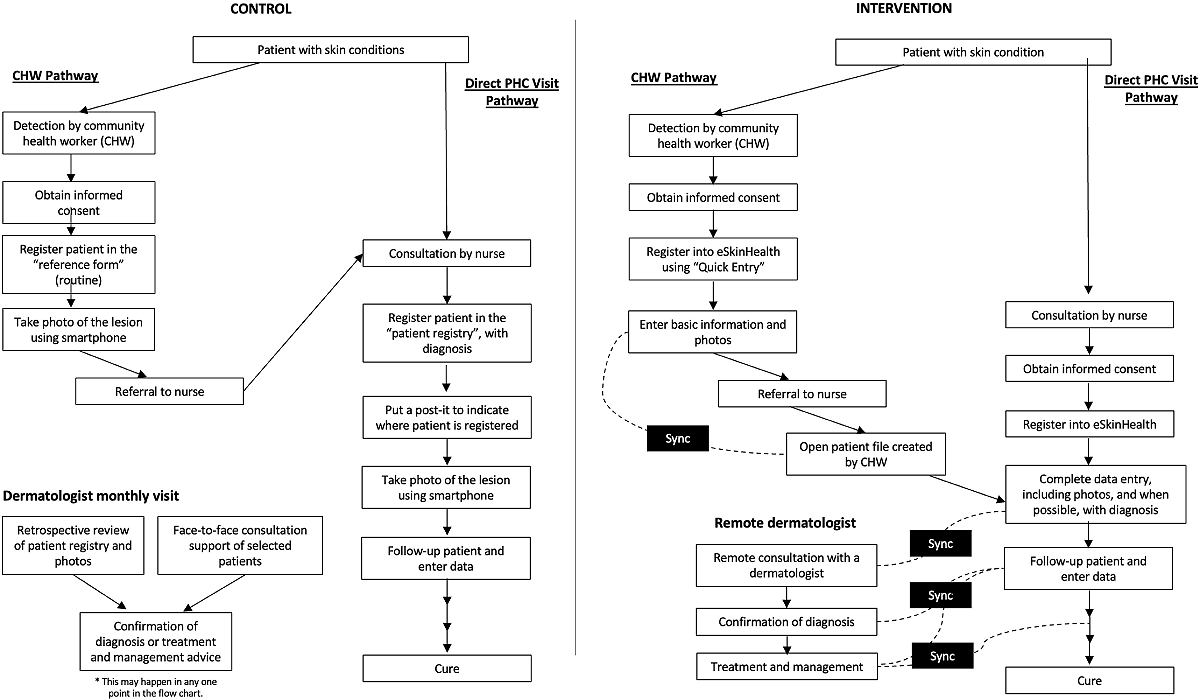
Patient flowchart for the 3-month pilot project of eSkinHealth app. CHW: community health care workers; PHC: primary health center.

This study involved the same local health care providers who participated in the usability study above. The patient flow for the trial is provided in Figure 2. The CHWs were instructed to register patients when they detected a suspicious case of skin NTDs or any skin conditions seemingly of importance and refer them to their designated PHCs. Patients suspected of having the targeted skin NTDs (Buruli ulcer, leprosy, lymphatic filariasis, scabies, and yaws) and other skin conditions who provided informed consent during consultation at PHC throughout the trial period were enrolled by the nurses and registered on the eSkinHealth app platform. The nurses were instructed to enter clinical data at the time of the initial visit and every follow-up until cure in the eSkinHealth app, including photos of skin lesions. The data were uploaded when connected to the internet and integrated into 1 database server. When they needed a consultation, a request was sent to remote dermatologists affiliated with the project, either in the cities of Abidjan or Bouaké, and advice or clinical confirmation was provided. Dermatologists were instructed to review registered cases periodically and provide their assessment and recommendations wherever possible.

As for the control arm, the number of cases with skin diseases was retrieved from the patient consultation registry booklet issued by the Ministry of Health and from the WHO skin NTDs reporting forms [25]. The CHWs were provided with paper reference forms and were instructed to refer any suspicious cases of skin NTDs or any skin conditions seemingly of importance to their designated PHCs. All participants in the control arm were also equipped with smart tablets or phones and were instructed to take photographs of skin lesions and create and organize them in an electronic folder labeled with patient names and IDs. All cases of skin NTDs were diagnosed and managed following the national standard guidelines.

For monitoring purposes, monthly field visits were made by the study team during the study period. During the visits, additional training on dermatology and, for the intervention arm, technical assistance on the usage of the app was provided. On request, face-to-face consultations with patients with skin conditions were performed.

### Outcome Measurement

For the usability study, we used a validated questionnaire, the SUS, to assess the usability of the app and portal. The outcome measurement was an average score of SUS. Bangor et al [32] found that the SUS was highly reliable (α=.91) and useful over a wide range of interface types [33,34]. The SUS consists of 10 statements with responses in the form of a 5-point Likert scale (eg, 1: strongly disagree; 5: strongly agree). According to Bangor and Miller [33], a SUS score above 68 would be considered above average. In addition, a SUS score above 80 is considered excellent and places the product in the top 10% of products tested [35]. We assessed the usability at baseline, the midpoint (6 weeks), and the end of the study (12 weeks). Semistructured, in-depth interviews were performed at the end of the study.

For the effectiveness study, differences in the number of cases diagnosed and followed up between the intervention group (diagnosis with the app) and the control group (usual care) were measured as the primary outcome.

### Data Collection

For the usability study, all survey data were obtained by study staff. Semistructured, in-depth interviews were all conducted in French, lasted between 10 and 20 minutes, and were held in private spaces either outside or inside the PHC or at the district hospital. All participants gave informed consent for voice-recording their interviews. All interviews were conducted by 2 staff members trained in and experienced in qualitative research. For the effectiveness study, the data collected using the eSkinHealth app were used.

### Analysis

Statistical analyses were performed using Stata software (version 16; StataCorp). The threshold for statistical analyses was set at *P*<.05 in a 2-tailed test. We summarized the baseline data by group assignment using descriptive statistics: means and SDs were used for continuous data with a normal distribution, medians and IQRs for skewed data, and percentages for categorical data. As for our primary outcome, we compared the number of patients diagnosed between the control and intervention groups using the chi-square test. Qualitative data were first transcribed and translated verbatim from French into English by a translator. They were then imported into the MAXqda software (version 20.4.2; VERBI GmbH) and coded (25 codes in total). Two researchers (RRY and AY) were responsible for coding all interviews. The frequency of the coded segments and relations between the segments were examined, resulting in identifying 6 main themes. All researchers checked for consistency, similarity, and diversity within and across groups and themes that we all agreed upon.

### Ethics Approval

The procedures of our methods have been reviewed and approved by the institutional review board (IRB) of the Ministry of Health, Côte d’Ivoire (No. IRB000111917) and by Tulane University (IRB 2020-2054-SPHTM). This study is registered at ClinicalTrials.gov (2020-2054). Informed consent was obtained in French, which is the official language of Côte d’Ivoire. In cases where a participant only spoke a local language, a member of our study team translated the informed consent form verbally to obtain consent. Our informed consent descriptions included consent for the primary data collection and secondary analyses of research data, including the use of images of skin lesions. No compensation was offered to patients, as the study’s aim was to support routine health care practices with minimal or no harm.

For storage of data registered in the eSkinHealth app, we have been using the Simple Storage Service (S3) storage of the Amazon Web Service server, which offers a safe, secure, highly durable storage infrastructure with continuous backups, regulated under the US Health Insurance Portability and Accountability Act. Only the study team and those registered with the eSkinHealth app system (nurses and CHWs) had access to the data. Access to data in eSkinHealth is regulated by user levels, meaning that, for example, nurses and CHWs can only view patient data designated to their PHC or catchment area. All paper documentation, including the signed consent forms, was stored in a secure cabinet, and access was available only to the study staff approved by the IRB. All data were deidentified during analysis.

## Results

Table 1 provides characteristics of local health care providers recruited to the study by each arm. Their mean ages were 40.5 and 42.5 years for the intervention and control arms, respectively, and all were male. Educational backgrounds differed for CHWs, but the distribution was the same between the 2 arms. All participants were familiar with and used cell phones or tablets on a daily basis, while the frequency of use of computers varied between individuals.

SUS was applied to 12 users of the eSkinHealth app in the intervention arm, and the results are shown in Table 2. The average scores at baseline, the midpoint (6 weeks), and the end of the study (12 weeks) were 72.3 (SD 11.5), 72.3 (SD 12.4), and 86.3 (SD 10.8), respectively.

The in-depth semistructured interviews were conducted with 20 participants from both arms: 5 nurses (3 from intervention, 2 from control) and 15 CHWs (7 from intervention, 8 from control). In addition, we invited 2 dermatologists and 2 program managers (district and national levels) for the interviews.

Two of the 3 nurses (66%) and 4 of the 7 CHWs (57%) in the intervention arm felt that it was initially a challenge to “manipulate” the app and also provide a diagnosis of skin lesions, and this challenge was overcome with time. The main difficulty faced with the app was over synchronization of data to the server due to poor internet connectivity in some study areas. Besides synchronization, no significant challenges or difficulties were mentioned.

The only problem I faced was the synchronization one...But with your [study team’s] support, finally we are able to manipulate easily. Now I go quicker in synchronizing. It used to take more time but now within three minutes I can synchronize.

CHW No 1-7, intervention

Concerning the smart tablets, 2 CHWs of the 10 individuals (20%) in the intervention arm complained that the tablet was “big and heavy.” One nurse and one CHW (20%) requested a flash function for the camera “because during the night it’s difficult to take photos” (our tablets did not have flash embedded in their cameras). One nurse (10%) pointed out that the quality of photographs taken with tablets was poorer compared to smartphones.

All 3 nurses in the intervention arm (100%) were satisfied with the app because they can be in contact with dermatology specialists when encountering cases that are difficult to diagnose by messaging them and getting support. For the CHWs, 5 out of 7 (71%) mentioned that they were satisfied with the project, and their satisfaction came from being equipped with a tablet and an app. It created self-confidence in their job and also supported their increasing reputation in their communities.

Really! With this tablet, it made my work as a CHW easier. With this tablet, I am well known in my working area. My practice improved because now I can use the device which was given to me. I can register and synchronize myself. Also, I can now stand before a patient and speak [feel more confident]. In the past, I could not stand before a patient and speak to him without tension. Before I got the tablet, I did some referrals [to PHCs] but it was not with insurance. Knowing that I have the tablet, I can refer more people with more insurance about what I am doing.

CHW No 1-4, intervention

There is a change [to my practice] because in the past we used to work without any guidance, but now that we have the tablets, everybody comes to us. Now we take their photos and we send. Sometimes they come to us and ask: “where is my photo you took last time”. When we open the tablet and show them the photos, then they trust us.

CHW No 1-11, intervention

One nurse and 2 CHWs (30%) mentioned the benefit of portability of the app. With the app, they had the mobility to go see patients in their communities.

We are no more obliged to transport our register to conduct diagnosis. Everywhere we have the tablet, we can consult patients.

Nurse No 1-9, intervention

**Table 1:** Characteristics of local health care providers in the study (N=12).

		Intervention		Control
**Age (years), mean (SD)**		40.5 (8.6)		42.5 (10.2)
	18-29, n (%)	1 (8.3)	0 (0.0)	
	30-39, n (%)	5 (41.7)	6 (50.0)	
	40-49, n (%)	4 (33.3)	3 (25.0)	
	50 years or older, n (%)	2 (16.7)	3 (25.0)	
**Sex, n (%)**				
	Male	12 (100.0)	12 (100.0)	
	Female	0 (0.0)	0 (0.0)	
**Educational background, n (%)**				
	Primary school	3 (25.0)	3 (25.0)	
	Junior high school	4 (33.3)	4 (33.3)	
	Senior high school	1 (8.3)	1 (8.3)	
	University or graduate school	4 (33.3)	4 (33.3)	
**Occupation, n (%)**				
	Community health worker	8 (66.7)	8 (66.7)	
	Nurse	4 (33.3)	4 (33.3)	
**Working periods, n (%)**				
	Less than 1 year	2 (16.7)	1 (8.3)	
	1-5 years	2 (16.7)	1 (8.3)	
	6-10 years	5 (41.7)	4 (33.3)	
	More than 10 years	3 (25.0)	6 (50.0)	
**Frequency of use of cell phones/tablets, n (%)**				
	Every day	12 (100.0)	12 (100.0)	
	A few times per week or less	0 (0.0)	0 (0.0)	
**Frequency of use of computers, n (%)**				
	Every day	3 (25.0)	3 (25.0)	
	A few times per week	1 (8.3)	1 (8.3)	
	A few times per month	0 (0.0)	0 (0.0)	
	A few times per year	1 (8.3)	1 (8.3)	
	Never	7 (58.3)	7 (58.3)	

**Table 2:** Results with System Usability Scale (SUS) scores (n=12)

System Usability Scale	Baseline, mean (SD)	Week 6, mean (SD)	Week 12, mean (SD)
I think that I would like to use this system frequently.	4.00 (0.00)	4.17 (0.94)	4.50 (0.80)
I found the system unnecessarily complex.	1.33 (0.65)	1.83 (1.53)	1.25 (0.87)
I thought the system was easy to use.	3.83 (0.72)	4.42 (0.67)	4.58 (0.79)
I think that I would need the support of a technical person to be able to use this system.	3.25 (1.82)	2.75 (1.77)	1.58 (1.38)
I found the various functions in this system were well integrated.	4.42 (1.17)	4.58 (0.79)	4.67 (0.65)
I thought there was too much inconsistency in this system.	1.25 (0.62)	2.25 (1.42)	1.25 (0.87)
I would imagine that most people would learn to use this system very quickly.	4.00 (1.35)	3.33 (1.56)	3.25 (1.77)
I found the system very cumbersome to use.	2.92 (1.51)	2.08 (1.56)	1.50 (1.24)
I felt very confident using the system.	4.42 (1.00)	3.75 (1.77)	4.67 (0.65)
I needed to learn a lot of things before I could get going with this system.	4.00 (1.48)	2.42 (1.68)	1.58 (1.38)
SUS score^a^	72.3 (11.5)	72.3 (12.7)	86.3 (10.8)

^a^Calculating SUS score: X = Sum of the points for all odd-numbered questions – 5; Y = 25 – Sum of the points for all even-numbered questions; SUS score = (X + Y) × 2.5.

All dermatologists and program managers interviewed (4/4, 100%) described the benefits of having the app to support nurses working in the very peripheral areas, allowing them to know the conditions of patients who are living hundreds of kilometers away, as most dermatologists are “concentrated in big cities.” They all voiced the opinion that it has enabled capacity building for those nurses and CHWs and strengthened their ability to screen, diagnose, and treat skin diseases.

The app enable us to be close to our patients. We are far physically but close to them virtually with this application.

Dermatologist No 1

All participants at all levels (24/24, 100%) felt the benefits of the project in general. All nurses, including those from the control arm (5/5, 100%), voiced that the project benefited them in providing a better and more accurate diagnosis rather than just writing down “dermatosis” when they see skin conditions, ultimately benefiting their patients. They showed appreciation for training them on basic skin diseases, not only the targeted skin NTDs. In addition, several participants (6/24, 25%) mentioned that they had observed behavioral changes in community members and that more people were coming out to disclose their skin diseases, which they used to hide before the project.

When you make an accurate diagnosis, it’s for the benefit of the patients. The patient are at the core of our activities. It’s the patient who gains. We, in return are satisfied.

Nurse No 1-12, intervention

With the training in dermatology, now in my village, I’m known as a dermatologist. This is already a success in my job.

Nurse No 2-1, control

Dermatology is an area that I love but since I am not equipped, I could not do well. The project is welcome because it enabled us to go deeper and to know new things. We learnt basic skin lesions which are at the beginning of dermatology. It enabled us to learn more and to treat more disease than the five targeted ones.

Nurse No 2-2, control

During the study period, a total of 207 and 311 patients with skin conditions were recruited in the intervention and control arms, respectively. In the intervention arm, their mean age was 31.9 (SD 22.3), and 37.2% were female. In the control arm, their mean age was 20.1 (SD 18.6), and 46% were female. The composition of skin diseases registered by intervention and control arms is provided in Tables 3 and 4. A significant difference was observed in the number of cases of skin NTDs between the intervention and control arms, that is, 79 and 17 cases, respectively (*P*=.002; Table 3). Moreover, when the number of cases of skin NTDs in which dermatologists either made or confirmed diagnosis was compared between the intervention and the control arms, the difference was further widened by almost 10-fold (*P*<.001). One case of mycetoma, which is a skin NTD not well documented in Côte d’Ivoire and was outside our target skin NTDs, was diagnosed in the intervention arm with the remote support of a dermatologist. Proportions of child and female cases with confirmatory diagnoses of skin NTDs in the intervention arm are provided in Multimedia Appendix 3. Besides the skin NTDs, more skin diseases and conditions were reported from the control arm than from the intervention arm (*P*<.001; Table 4). However, 100 cases (66%) were not given any particular diagnosis or description of a condition in the control arm and were documented only as a “dermatosis.” For a total of 320 cases that were registered from both intervention and control arms, 53 different diagnoses and conditions were indicated. This list is provided as Multimedia Appendix 4.

In the intervention arm, a total of 151 cases (72.9%) were diagnosed on the eSkinHealth platform. The median number of days from entry to diagnosis by remote dermatologists was 21 (IQR 25%-75%, 16-63) and the average was 34.9 (SD 25.5) days. The diagnosis of 54 cases (26.1%) was supported on-site by dermatologists during field visits due to any reason, including upload failure of images to the server, at the time of reviewing the patient file. Three cases in the intervention arm were diagnosed from the results of a skin biopsy performed during the field monitoring visit (lichen planus, pyogenic granuloma, and atheroma). In the control arm, the diagnosis of 20 patients (6.4%), among whom 3 patients had skin NTDs, was confirmed by dermatologists during the monitoring field visit; 11 patients (3.5%) were referred to the district general hospital; the rest were nonconfirmed.

Results on the comparison of diagnoses by nurses in the intervention arm against those by dermatologists are shown in Table 5. The overall diagnosis rate of skin NTDs by nurses was 62%, with the highest for yaws (with the use of a rapid diagnostic kit) followed by scabies.

**Table 3:** Number of cases of skin diseases, intervention versus control—skin-related neglected tropical diseases (skin NTDs).

	Intervention					Control					Total cases confirmed, n (%)
	Confirmed^a^	Suspected	Subtotal	Confirmed cases among all cases, %	Confirmed^a^		Suspected	Subtotal	Confirmed cases among all cases, %		
Buruli ulcer	26	0	26	100	1		2	3	33.3	29 (93.1)	
Leprosy	11	0	11	100	1		0	1	100	12 (100)	
Lymphatic filariasis	4	0	4	100	0		0	0	N/A^b^	4 (100)	
Mycetoma	1	0	1	100	0		0	0	N/A	1 (100)	
Scabies	34	0	34	100	0		6	6	0	40 (85.0)	
Yaws	3	0	3	100	6		1	7	85.7	10 (90.0)	
Total	79	0	79	100	8		9	17	47.1	96 (88.5)	

^a^Includes cases either diagnosed or confirmed by dermatologists.

^b^N/A: not applicable.

**Table 4:** Number of cases of skin diseases, intervention versus control—skin diseases and conditions besides skin-related neglected tropical diseases (skin NTDs).

	Intervention	Control	Total
Registered with diagnosis or condition^a^, n	126	194	320
Registered only as “dermatosis,” n	2	100	102
Total, n	128	294	422
With diagnosis or condition, %^a^	98.4	66.0	74.6

^a^Includes both confirmed and suspected diagnosis.

**Table 5:** Number of cases of skin-related neglected tropical diseases (skin NTDs) diagnosed by nurses and additionally diagnosed by dermatologists.

Disease	Cases diagnosed by nurses, n	Additional diagnoses by dermatologists, n	Diagnosis rate by nurses, %
Buruli ulcer	13	13	50.0
Leprosy	4	7	36.4
Lymphatic filariasis	2	2	50.0
Mycetoma	0	1	0
Scabies	27	7	79.4
Yaws	3	0	100
Total	49	30	62.0

Among all registered patients (n=207) in eSkinHealth, 59 cases (28.5%) were detected by the CHWs and were referred to the nurses. For the control arm, this was unclear as instructions on using referral paper forms were not followed. Alternatively, photographs of skin lesions were taken for 92 patients and 58 patients by CHWs and nurses using smartphones or tablets provided to them, respectively. Through reviewing these 2 image sets, at least five cases were photographs of the same patients, suggesting that these cases were referred from CHWs to nurses. Although the images were taken, the participants in the control arm did not organize the photographs with patient information as instructed, and further assessment of the images was therefore not possible, resulting in a high number of non-confirmed cases.

In the intervention arm, 24 of 207 patients (11.6%) had 1 follow-up (this excludes the first visit), 11 patients (5.3%) had 2 follow-ups, and 3 patients (1.4%) had 3 follow-ups entered by the nurses. This data was not available for the control arm.

## Discussion

### Principal Findings

This paper reported on a 3-month pilot trial to evaluate the usability and effect of the eSkinHealth app for the early detection and management of skin NTDs and other skin conditions in remote communities in sub-Saharan Africa. This app has been our invention, and to our knowledge, there is currently no other mHealth app of the kind that is developed for the collection of clinical data on skin diseases that can be used both online and offline, making it fit for use in LMICs. We found that our app was appreciated by most users, as shown by the high SUS scores as well as our interview results. On the other hand, while the app is developed to be capable of offline usage, the difficulty was still felt with the poor internet connectivity when uploading and retrieving data.

While our SUS scores were rated “excellent” for all 3 time points, they significantly increased during the intervention, jumping from a mean of 72.3 both at baseline and week 6 to 86.3 at the end of the project. This could be showing that the time needed to get accustomed to the system was around 2 to 3 months. We do not necessarily consider this to be overly long, as the usage of the app involves a behavioral change in their daily practice, and usually behavioral change takes time [36]. This being the case, we still need to make efforts to improve our system to increase usability and feasibility. In addition, although one of the major functions and expectations of the app was for it to be used for following up on patients, only a limited proportion of patients (18.3%) had more than one follow-up. While not enough time has elapsed for most patients to require follow-up and to assess this, it may also be an indication that this function was not well understood by our participants. Moreover, when participants were asked why follow-up data were not taken, oftentimes their replies were that “patients did not come back.” Losses in follow-up rates in LMICs, especially in sub-Saharan Africa, for any condition are known to be high due to multiple factors [37-39]. There is potential for mHealth tools and telemedicine systems to fill this gap [40].

The benefits of using the app were shown by the higher number of targeted skin NTDs diagnosed in the intervention arm as compared to the control arm. However, it was interesting to see that more patients with skin diseases and conditions were documented in the control arm, which may be demonstrating the high prevalence of skin diseases in these communities, supporting previous reports [1-7]. Or, it could also be that with the decision-support provided by the eSkinHealth app, the patients seen by providers in the intervention arm were more likely to be screened out. Although we did not explicitly hear about the workload of entering data into the eSkinHealth app, the lower number of cases in the intervention arm for the overall number of skin diseases could possibly also be explained by the felt need for extra time in seeing a patient and entering their data in the system. During this pilot study, we did not measure the time required to register each patient, which should be considered for our future studies. Nevertheless, while there were more patients registered in the control arm, one-third of them were only documented as “dermatosis” compared to only 2 undiagnosed cases in the intervention arm, which was a significant change in their practice between the 2 arms; with the intervention, patients were now provided with their diagnosis. This was also described by our participants during their interviews.

Some users of the app voiced difficulties with “synchronization,” or the uploading or retrieving of data. This was mainly due to 2 factors: the location of the users and the data size of the images. The issue was mostly experienced by the same users living in more remote rural areas with poor network connectivity. Poor or no connection to network services has been one of the major challenges shared across projects aiming at implementing mHealth strategies in LMICs [19,41,42]. In our study, sometimes changing the network service improved their synchronization. Nonetheless, most times, text data were able to be transmitted to the server, but the major challenge was with images, as they require a larger bandwidth. This limited the remote diagnosis by dermatologists and therefore had a considerable impact on the overall project outcome. The size of each image was 2080 × 1560 pixels. There is no universally accepted standard for image sizes for teledermatology, and recommendations vary between guidelines and reports [43-45]. On the other hand, there is a report that states that a minimal image size can be 768 × 512 pixels, and above this does not substantially improve the results [46]. Therefore, 1 option for us is to decrease the size of the images to make them more transmissible, but this requires careful consideration. This may limit us, for example, in the accuracy of diagnosis and future usage of the collected images. Although there is ever-increasing connectivity globally, there are still challenges to overcome in the settings of LMICs, and there is a constant need to think about how we can overcome them.

Dermatologists supported 27% of cases in the intervention arm for diagnosis on-site during monitoring visits. One reason for this was the absence of images owing to the abovementioned issue of synchronization, and images were absent for making remote consultations. Or sometimes, images taken by local health care providers were of poor quality to make a diagnosis. There was no function where dermatologists could indicate these issues during the time of this pilot project, and therefore, unfortunately, we were unable to calculate the number of cases that fell into these categories. This is currently being addressed. Yet another reason for an on-site diagnosis was due to the nature of the disease. Subcutaneous conditions such as atheroma and lipoma are difficult to diagnose remotely through regular photographic images, and these conditions tended to be diagnosed on-site. This observation is supported by previous studies showing that teledermatology with regular photographs is useful for superficial lesions but is limited otherwise [47].

Indeed, we observed definite advantages of using the app and the platform established around it in the management of skin diseases at our study sites compared to not using it. A timely and accurate diagnosis of both skin NTDs and other locally prevalent skin diseases has potential benefits beyond what can be quantified during the brief period of this study’s implementation. These can include the prevention of disabilities and a better understanding of disease epidemiology. It is also a very scalable intervention that would ultimately leverage scarce in-country dermatologists and international dermatologists to supplement and complement local health care workers. We also observed that our participants improved their knowledge of dermatology over time while using the app, which we plan to measure in our future project. It is a limitation of this study that we were unable to provide accurate data for the control arm, while simultaneously representing the advantages of using the app. While we did train our participating providers in the control arm to take photographs and organize them so that we could compare the 2 arms, this was not pursued adequately to make valid comparisons between the 2 arms. Involving CHWs in our approach was effective, as they are the ones who are the closest to patients, as has also been demonstrated in previous studies [42]. We observed that the referral pathway created by the eSkinHealth app worked, with close to 30% of cases diagnosed being those that were connected from CHWs to PHCs. If CHWs can be empowered and involved more, for instance by being equipped with the app, this may contribute to the control of skin diseases, including skin NTDs, to a large extent. In a systematic review that investigated involving CHWs in the delivery of primary health care services through the use of mHealth tools, it was found that not only did mHealth tools change how CHWs delivered care, but they also led to new forms of positive engagement and relationships with people in their communities, creating self-confidence [42]. Similar findings were observed among the CHWs in our study.

Through our study, one case of mycetoma, which is listed as one of the skin NTDs but was outside of our target diseases, was identified. This was because we were unaware that the disease existed at our study sites. This indicates that diseases such as mycetoma are being missed and remain unreported due to a lack of awareness. Moreover, 6 cases of snakebites were reported from the control arm. Snakebite is not a disease grouped as a skin NTD, but it is one of the NTDs recently added to the WHO NTD list in 2017. While its occurrence and management differ largely from those grouped as skin NTDs, snakebites also manifest as skin lesions and, therefore, can potentially be part of an integrated approach for skin NTDs. It is important that efforts are made to gain a better understanding of the epidemiology, ideally in combined efforts to save resources and time, to support the implementation of effective and efficient disease control measures for these diseases [14]. Our integrated training on dermatological conditions not limited to skin NTDs was highly welcomed by local health care providers, and this kind of approach should be promoted with relevant diseases in the community.

A report of some conditions that were not part of the set menu of skin diseases included in the eSkinHealth app allowed us to consider further improvements. Fifteen cases of sexually transmitted diseases (STDs) (5 females and 10 males) were reported from the control arm and one (secondary syphilis) from the intervention arm, which was a critical finding of this study. Although we do not know their exact diagnosis, this result indicated that this set of diseases existed in the communities but was not registered in eSkinHealth. This may also imply that it is difficult to obtain patient consent for registering them on the digital platform and taking photographs of their private body parts. Meanwhile, patients with hydroceles and hernias involving male genital areas were registered, and so it is suspected that it is not only about the body parts that patients become hesitant about disclosing their disease. There is much stigma and discrimination around STDs, and their disease burden in these communities is largely hidden [48-50]. This needs to be addressed in some way or other, however, which may be a difficult area for a digital health tool to intervene in.

### Limitations

We acknowledge several limitations of our study. First, although the app can be used offline, an internet connection is required for certain functions. Poor internet connectivity affected our results and hindered the assessment of its true effectiveness. For instance, some patients underwent diagnosis on-site by dermatologists rather than through the app during our monitoring visits. Furthermore, some of the benefits of the project outlined here may have come from the monitoring visits that were made by the study team and therefore cannot solely be attributed to the effectiveness of the app. However, it was important that we paid these monitoring visits to support our participants in using the system and to follow our study protocol. Second, all our participants were male. Usually, nurses working in PHCs in these remote communities are mostly male because females tend not to volunteer to be placed in such areas as it involves some duties traditionally not done by women in this setting, such as traveling to difficult-to-reach villages on motorbikes. Some diseases may have been missed due to this gender imbalance among our participants, such as STDs in females. Lastly, this is a pilot trial involving 8 PHCs in 1 health district, and therefore, the study findings may not be generalizable to other health districts in Côte d’Ivoire. We believe that additional research is needed to further evaluate the usability and effectiveness of the eSkinHealth app, taking the learnings from this pilot study into account as it launches and becomes more widely used.

### Conclusions

The burden of skin diseases in LMICs is often neglected, as many are not considered fatal. It is in LMICs and marginalized communities that mHealth tools could be most useful and valuable [40]. This study examined the usability and effectiveness of the eSkinHealth app to improve the early detection and case management of skin NTDs and other skin conditions, which provided promising results. It is also portable, and therefore, it can be used in remote communities in LMICs where infrastructure is usually very poor. We plan to further test our app in a wider region of sub-Saharan African countries. Furthermore, given the importance of improving the early detection and case management of skin NTDs in LMICs, our study results provide a compelling rationale for infectious disease policymakers and decision makers regarding mHealth interventions for skin NTDs in these settings.

## Appendix

Multimedia Appendix 1 System Usability Scale (French version) used in the study.

Multimedia Appendix 2 Topic guidelines fro semistructured in-depth interviews.

Multimedia Appendix 3 Number of child and female cases of skin-related neglected tropical diseases confirmed in the intervention arm.

Multimedia Appendix 4 Skin diseases and conditions reported besides skin-related neglected tropical diseases (intervention vs. control).

## Abbreviations

CHW: community health care worker
IRB: institutional review board
LMIC: low- and middle-income country
mHealth: mobile health
NTD: neglected tropical disease
PHC: primary health center
Skin NTD: skin-related neglected tropical disease
SUS: System Usability Scale
WHO: World Health Organization

## Data Availability

The data sets generated or analyzed during this study are available from the corresponding author on reasonable request.
