# Supplementary material for: An mHealth App (eSkinHealth) for Detecting and Managing Skin Diseases in Resource-Limited Settings: Mixed Methods Pilot Study
Source: JMIR Dermatol. 2023 Jun 14;6:e46295. doi: 10.2196/46295 (PMC10335127; doi:10.2196/46295)
Supplement: Multimedia Appendix 1 [file derma_v6i1e46295_app1.pdf]

NOM DU PARTICIPANT: DATE:

System Usability Scale

Pour chacune des affirmations suivantes, veuillez cocher une case qui décrit le mieux vos réactions à eSkinHealth aujourd'hui.

		Pas du tout d'accord				Tout à fait d'accord
1.	Je pense que je vais utiliser eSkinHealth fréquemment.	1	2	3	4	5
2.	Je trouve eSkinHealth inutilement complexe.	1	2	3	4	5
3.	Je pense que eSkinHealth est facile à utiliser.	1	2	3	4	5
4.	Je pense que j'aurai besoin de l'aide d'un technicien pour être capable d'utiliser eSkinHealth.	1	2	3	4	5
5.	J'ai trouvé que les différentes fonctions de eSkinHealth ont été bien intégrées.	1	2	3	4	5
6.	Je pense qu'il y a trop d'incohérence dans eSkinHealth.	1	2	3	4	5
7.	J'imagine que la plupart des gens serait capable d'apprendre à utiliser eSkinHealth très rapidemen.	1	2	3	4	5
8.	J'ai trouvé eSkinHealth trés lourd à utiliser.	1	2	3	4	5
9.	Je me sentais très en confiance en utilisant eSkinHealth.	1	2	3	4	5
10.	J'ai besoin d'apprendre beaucoup de choses avant de pouvoir utiliser eSkinHealth.	1	2	3	4	5

Commentaires (optionnel):
