# Supplementary material for: An mHealth App (eSkinHealth) for Detecting and Managing Skin Diseases in Resource-Limited Settings: Mixed Methods Pilot Study
Source: JMIR Dermatol. 2023 Jun 14;6:e46295. doi: 10.2196/46295 (PMC10335127; doi:10.2196/46295)
Supplement: Multimedia Appendix 2 [file derma_v6i1e46295_app2.pdf]

Supplementary file 2. Topic guidelines for semi-structured in-depth interviews

1. Introduction

2. What do you think about the project?
   - Strength
   - Weakness

3. What were the challenges?
   - Did you face any challenges?
   - If yes, what were they?
   - How often did you face them?

4. How do you think your practice has changed?
   - Improvement? If so, how?
   - No change? If so, how?
   - Worsened? If so, how?
   - Explain about pre- and post-intervention.

5. How was the acceptance by patients and their families of the project?
   - Good? If so, what do you think were the reasons?
   - Poor? If so, what do you think were the reasons?
   - Did you face any denial of patients and their families to be registered using the eSkinHealth app? If yes, please explain.

6. What will be your recommendation for the project? How can the project improve?

7. Any other points participants would like to discuss.
