# Supplementary material for: An mHealth App (eSkinHealth) for Detecting and Managing Skin Diseases in Resource-Limited Settings: Mixed Methods Pilot Study
Source: JMIR Dermatol. 2023 Jun 14;6:e46295. doi: 10.2196/46295 (PMC10335127; doi:10.2196/46295)
Supplement: Multimedia Appendix 3 [file derma_v6i1e46295_app3.pdf]

Supplementary file 3. Number of child and female cases of skin NTDs confirmed in the intervention arm

	**Total no. of cases diagnosed**	**Child (< 15 yrs)**	**% child (< 15 yrs)**	**Female**	**% female**
Buruli ulcer	26	10	38.5%	11	42.3%
Leprosy	11	1	9.1%	1	9.1%
Lymphatic filariasis	4	1	25.0%	3	75.0%
Mycetoma	1	0	0%	0	0%
Scabies	34	21	61.8%	21	61.8%
Yaws	3	1	33.3%	2	66.7%
**TOTAL**	**79**	**34**	**43.0%**	**38**	**48.1%**
