# Supplementary material for: An mHealth App (eSkinHealth) for Detecting and Managing Skin Diseases in Resource-Limited Settings: Mixed Methods Pilot Study
Source: JMIR Dermatol. 2023 Jun 14;6:e46295. doi: 10.2196/46295 (PMC10335127; doi:10.2196/46295)
Supplement: Multimedia Appendix 4 [file derma_v6i1e46295_app4.pdf]

Supplementary file 4. Skin diseases or conditions reported besides skin NTDs, intervention vs. control

	Intervention	Control	TOTAL
Abscess	3	9	12
Acne	1	0	1
Allergy	0	14	14
Anthrax	0	1	1
Bullous dermatosis	0	1	1
Candidiasis	0	5	5
Chancroid	0	1	1
Cellulitis	1	0	1
Contact dermatitis	4	0	4
Cyst (unspecified)	2	2	4
Cyst (atheroma)	2	1	3
Cyst (synovial cyst)	2	0	2
Dermatitis / eczema / skin eruption	5	5	10
Dry skin	1	0	1
Dyshidrosis with secondary infection	1	0	1
Epidermal carcinoma	2	0	2
Erysipelas	9	3	12
Fibrosis	1	0	1
Folliculitis	2	0	2
Furoncle	0	13	13
Gonorrhea	0	1	1
Herpes zoster	1	1	2
Hydrocele (non-LF)	5	1	6
Hypopigmented macule	0	1	1
Inguinal hernia	10	8	18
Impetigo	7	0	7
Lichen planus	4	0	4
Lipoma	27	8	35
Lupus	3	0	3
Lymphoedema post-erysipelas	1	2	3
Measles	0	4	4
Molluscum contagiosum	1	0	1
Mycosis	0	4	4
Nappy rash	0	1	1
Necrotizing fasciitis	11	2	3
Neurofibromatosis type 1	1	0	1
Onychomycosis	0	1	1
Paronychia	0	9	9
Pigmented macule	0	1	1
Pruritis	0	2	2
Pyogenic granuloma	1	0	1
Sexual transmitted diseases (STDs) (non-specified)	0	15	15
Snake bite	0	6	6
Steven Johnson’s Syndrome	1	0	1
Syphilis	1	0	1
Tinea	7	5	12
Tumor	1	0	1
Urticaria	0	3	3
Varicella	2	44	46
Verruca vulgaris	1	0	1

Viral infection	1	0	1
Vitiligo	3	3	6
Wound	1	17	18
**Sub-total**	**126**	**194**	**320**
‘Dermatosis’	2	100	102
**TOTAL**	**128**	**294**	**422**
